# FIT2 proteins and lipid droplet emergence, an interplay between phospholipid synthesis, surface tension, and membrane curvature

**DOI:** 10.3389/fcell.2024.1422032

**Published:** 2024-05-30

**Authors:** Juliette Graff, Roger Schneiter

**Affiliations:** Department of Biology, University of Fribourg, Fribourg, Switzerland

**Keywords:** lipid metabolism, Kennedy pathway, inositol auxotroph, metabolic disorders, membrane dynamics, lipid droplet morphology

## Abstract

Lipid droplets (LDs) serve as intracellular compartments primarily dedicated to the storage of metabolic energy in the form of neutral lipids. The processes that regulate and control LD biogenesis are being studied extensively and are gaining significance due to their implications in major metabolic disorders, including type 2 diabetes and obesity. A protein of particular interest is Fat storage-Inducing Transmembrane 2 (FIT2), which affects the emergence step of LD biogenesis. Instead of properly emerging towards the cytosol, LDs in FIT2-deficient cells remain embedded within the membrane of the endoplasmic reticulum (ER). *In vitro* studies revealed the ability of FIT2 to bind both di- and triacylglycerol (DAG/TAG), key players in lipid storage, and its activity to cleave acyl-CoA. However, the translation of these *in vitro* functions to the observed embedding of LDs in FIT2 deficient cells remains to be established. To understand the role of FIT2 *in vivo*, we discuss the parameters that affect LD emergence. Our focus centers on the role that membrane curvature and surface tension play in LD emergence, as well as the impact that the lipid composition exerts on these key parameters. In addition, we discuss hypotheses on how FIT2 could function locally to modulate lipids at sites of LD emergence.

## 1 Introduction

Lipid droplets (LDs) serve as specialized compartments for the storage of metabolic energy in the form of neutral lipids, such as triacylglycerol (TAG) and steryl esters (STE). Beyond their role in neutral lipid storage, LD formation is crucial for apparently unrelated processes, including the alleviation of stress responses, the regulation of membrane homeostasis, and the control of protein storage and degradation (Roberts and Olzmann, 2020; Zadoorian et al., 2023; Mathiowetz and Olzmann, 2024). Anomalies in LD formation is associated with several disorders, including metabolic syndromes like non-alcoholic fatty liver disease, pancreatic diseases, and obesity (Gluchowski et al., 2017; Hotamisligil, 2017; Zadoorian et al., 2023). Dysfunctional LD formation is also connected to non-metabolic syndromes such as neurodegeneration and bacterial infection (Ralhan et al., 2021; Safi et al., 2023; Zadoorian et al., 2023; Windham et al., 2024). A comprehensive understanding of the processes that coordinate and regulate LD formation is essential for establishing the causal relation between deficient biogenesis and the observable disorders.

LDs have their origin in the membrane of the endoplasmic reticulum (ER). The synthesis of neutral lipids relies on diacylglycerol (DAG) or free sterols and acyltransferases, resulting in the formation of TAG and STE, respectively. These neutral lipids accumulate between the ER leaflets, creating an oily lens, a process known as nucleation. As neutral lipid synthesis progresses, this lipid lens expands and buds towards the cytosol, ultimately developing into a mature LD. These emerged LDs remain connected to the ER membrane in both yeast and mammalian cells (Jacquier et al., 2011; Wilfling et al., 2013; Cottier and Schneiter, 2022).

The biogenesis of LDs is governed by numerous proteins, with seipin serving as an illustrative example. Seipin oligomers gather at ER-LD junctions, forming a ring of hydrophobic helices that stabilizes the incorporation of TAG into LDs (Schneiter and Choudhary, 2022; Salo, 2023). Cells lacking seipin have clustered LDs or a unique supersized LD. Similar to seipin’s role in regulating LD size and number, the Fat storage-Inducing Transmembrane 2 (FIT2) protein is important for controlling LD emergence (Kadereit et al., 2008).

The FIT protein family is conserved across eukaryotes and comprises two family members, FIT1 and FIT2, both of which have been implicated in LD biogenesis (Kadereit et al., 2008). FIT2 is expressed in most mammalian tissues, with higher levels in adipose tissue, while FIT1 expression is confined to skeletal muscles and heart tissues (Kadereit et al., 2008). FIT2 homologs are found in most eukaryotes, making it the most extensively studied protein within the FIT family (Kadereit et al., 2008). Deficiency in FIT2 is linked to metabolic disorders such as type 2 diabetes, as well as non-metabolic disorders like deafness-dystonia syndrome (Cho et al., 2011; Miranda et al., 2014; Zazo Seco et al., 2017). In mammalian cells, the absence of FIT2 results in a reduction in both the number and size of LDs (Kadereit et al., 2008).

An important phenotype associated with FIT2 dysfunction, conserved from yeast to humans, is the embedding of LDs in the ER membrane (Choudhary et al., 2015). *In vitro* studies indicate that FIT2 binds both TAG and DAG (Gross et al., 2011), and catalyzes the cleavage of acyl-CoA to acyl 4′-phosphopantetheine (Becuwe et al., 2020). This acyl 4′-phosphopantetheine is further dephosphorylated into acyl pantetheine, although the phosphatase responsible for this reaction and the biological function of the resulting acyl pantetheine have yet to be discovered (Becuwe et al., 2020). However, as of now, none of these functions have been confirmed *in vivo*. Consequently, the mechanisms through which FIT2 facilitates LD emergence remain to be fully understood. The objective of this review is to describe the factors that affect LD emergence, providing the ground to discuss possible functions of FIT2 proteins in this process.

The yeast *Saccharomyces cerevisiae* has two FIT2 homologs, Scs3 and Yft2, both harbor the two highly conserved histidine residues implicated in acyl-CoA cleavage *in vitro* (Hayes et al., 2017; Becuwe et al., 2020). Remarkably, even prior to the identification of the FIT protein family, Scs3 was recognized as necessary for the growth of yeast cells on inositol-depleted medium (Hosaka et al., 1994). Deletion of *SCS3* leads to a mild growth defect, exacerbated by the addition of choline to the medium. A similar growth phenotype is observed when the conserved histidine residues of Scs3 are mutated (Hayes et al., 2017; Becuwe et al., 2020). Thus, these histidine residues establish a link between the acyl-CoA diphosphatase activity of human FIT2 and the inositol auxotrophy of yeast mutants lacking Scs3 function. Consequently, comprehending the inositol auxotrophy of *SCS3*-depleted cells could be key to understand the function shared by FIT2 homologs *in vivo*.

## 2 All roads lead to phosphatidylcholine

**FIGURE 1:**
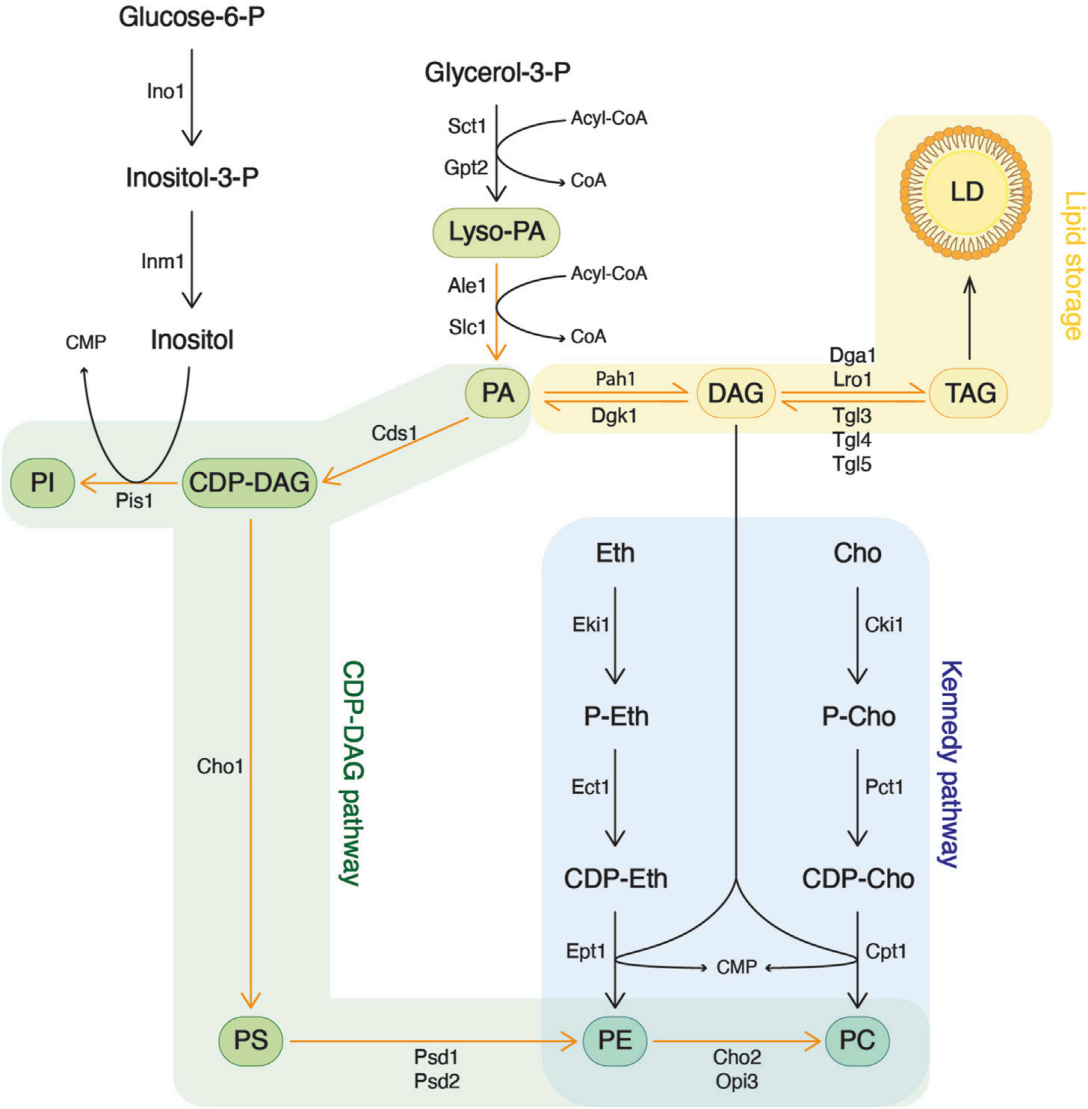
Phospholipid synthesis in the yeast *Saccharomyces cerevisiae*. Phospholipids originate from glycerol-3-phosphate, which is acylated to yield phosphatidic acid (PA). PA can then serve either for the synthesis of more complex phospholipids or can be converted to diacylglycerol (DAG) for lipid storage. Phosphatidylethanolamine (PE) and phosphatidylcholine (PC) can be synthesized through two different pathways. In the CDP-DAG pathway, phosphatidylserine (PS) is decarboxylated into PE, which is then methylated to form PC. On the other hand, the Kennedy pathway utilizes exogenous ethanolamine or choline, transferring it onto DAG, derived from the lipid storage pathway, to produce PE and PC, respectively.

Lipids are primary components of biological membranes, particularly phospholipids derived from the modification of phosphatidic acid (PA) (Figure 1). PA plays a pivotal role in determining the equilibrium between lipid storage, through DAG and TAG production, and membrane biogenesis through phospholipid synthesis (Sorger and Daum, 2003; Henry et al., 2012). PA is structured around a glycerol-3-phosphate backbone that undergoes two acylation events. The phosphate residue can then receive inositol to form phosphatidylinositol (PI) or serine to form phosphatidylserine (PS). Subsequently, PS can undergo decarboxylation to generate phosphatidylethanolamine (PE), which, upon three methylation steps, is converted to phosphatidylcholine (PC). This pathway of phospholipid biosynthesis is known as the CDP-DAG pathway or the *de novo* pathway.

An alternative route for PE and PC synthesis is provided by the Kennedy pathway, which utilizes exogenous ethanolamine and choline (Gibellini and Smith, 2010; McMaster, 2018). In this route, choline is first phosphorylated by choline kinase (Cki1 in yeast, CHKA/CHKB in mammals). Subsequently, phosphocholine is converted to CDP-choline by phosphocholine cytidylyltransferase (Pct1 in yeast, PCT1A/PCT1B in mammals). Finally, the activated choline is transferred onto DAG by choline phosphotransferase (Cpt1 in yeast, CHPT1 in mammals) to form PC. Analogous reactions occur with ethanolamine. When exogenous choline is present, the Kennedy pathway becomes the primary source of PC and is essential for most eukaryotic organisms (McMaster, 2018).

Every cellular membrane has its unique lipid composition (Schneiter et al., 1999; Harayama and Riezman, 2018). Each lipid species possesses unique physicochemical properties, including its dynamic shape, size, and electrostatic charge. Different lipid compositions are associated with various cellular functions. The specificity in composition influences the properties of membranes, affecting interactions both between lipids and with proteins. A particular combination of lipids may recruit specific proteins to carry out distinct cellular functions. For example, in yeast cells, lipid synthesis is regulated by the transcriptional repressor Opi1 and is dependent on the availability of inositol (Loewen et al., 2004; Hofbauer et al., 2018). When inositol is present, Opi1 localizes to the nucleus where it represses genes needed in inositol and lipid synthesis. However, upon inositol depletion, less PI is synthesized, resulting in an increase in PA levels in the ER. This prompts Opi1 to exit the nucleus and bind to the ER membrane through a direct interaction with PA. This sequestration of Opi1 at the ER membrane in turn de-represses the expression of genes for inositol and phospholipid synthesis, thereby promoting cell growth.

The hydrophobic core of LDs is covered by a monolayer having a unique composition of phospholipids derived from the ER membrane (Tauchi-Sato et al., 2002). This monolayer is predominantly composed of PC, PI, and PE, which together constitute about 88% of LD phospholipids. Conversely, it contains only low levels of PS, dimethyl-PE, and PA (Leber et al., 1994). The composition of phospholipids at ER domains from where LDs are formed is likely a primary factor regulating their proper emergence, as this process is affected by the physicochemical properties of membrane lipids. Indeed, phospholipids play a crucial role in influencing both membrane curvature and surface tension, which are two essential parameters controlling LD budding.

## 3 The phospholipid composition affects surface tension and LD emergence

**FIGURE 2:**
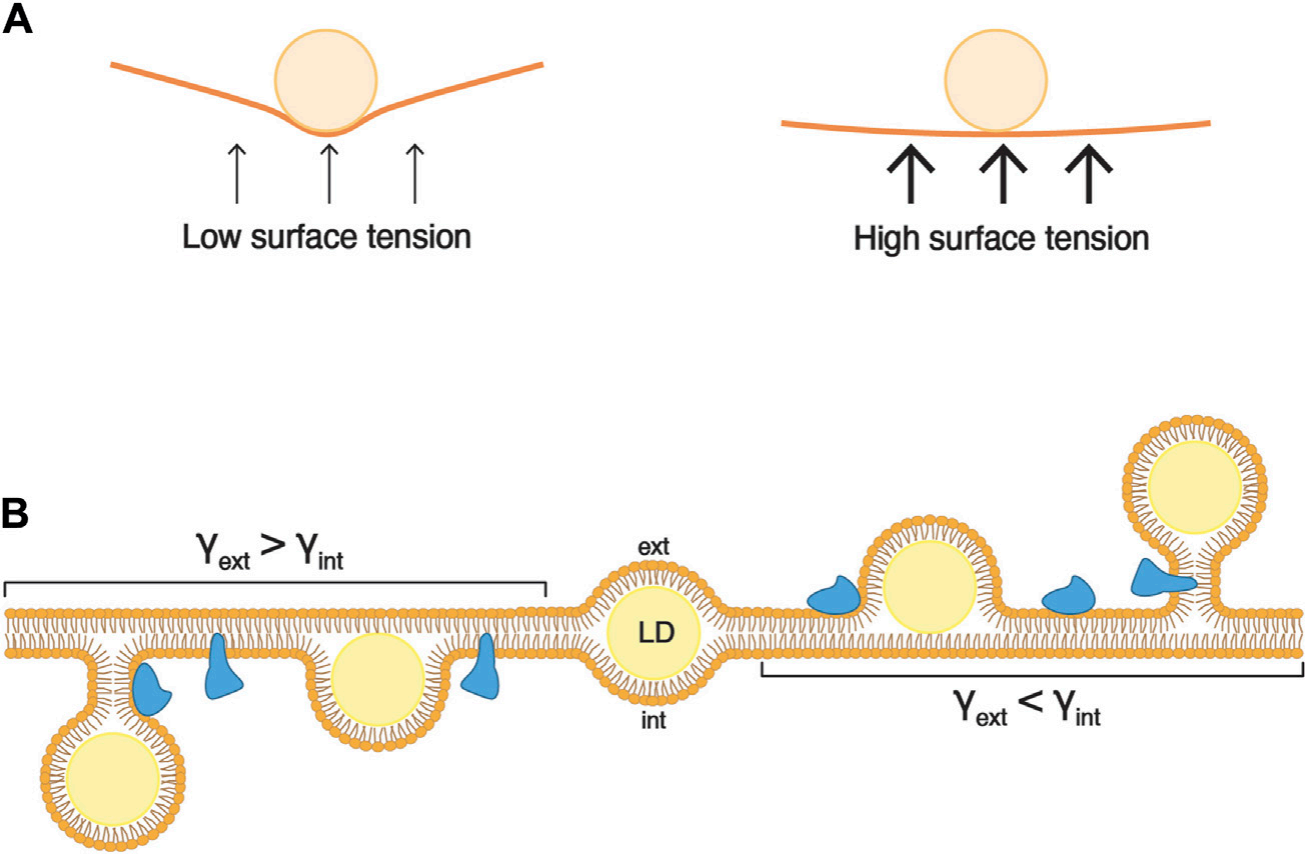
Asymmetry of surface tensions dictates the direction of LD budding. **(A)** Surface tension is the capacity of a surface to withstand an external force. For instance, if a ball is dropped onto a loosely stretched cloth (low surface tension), the force from the ball will deform the shape of the cloth. Conversely, if the cloth is tightly stretched (high surface tension), it will readily resist the force from the ball, and its shape will undergo minimal alteration. **(B)** During the formation of an LD, the expanding hydrophobic core (in yellow) exerts force on both leaflets of the ER bilayer. When the surface tensions (γ) of two layers are comparable, the LD remains uniformly embedded between the leaflets (center). Conversely, when the surface tension of one ER leaflet is lower than that of the other, the neutral lipid core is directed towards the leaflet with lower tension. The accompanying change in membrane curvature can be relieved by lipid synthesis and/or the insertion of proteins (in blue).

Surface tension refers to the ability of molecules at a liquid interface to withstand external forces (Figure 2A). This property primarily hinges on the interactions among the molecules constituting the liquid interface and is also affected by the quantity of molecules forming the surface (surface coverage). An area with high surface coverage tends to have lower surface tension compared to the same surface with lower surface coverage. Likewise, for two surfaces with identical surface coverage, the surface formed by molecules with strong interactions has higher surface tension than a surface formed by molecules interacting weakly with each other.

Since lipid layers act as separators between hydrophobic and aqueous environments, they are also subject to the principles of surface tension. In a vesicular bilayer, there are two distinct surface tensions to consider, one for each leaflet. The internal surface tension (γ_int_) pertains to the luminal leaflet, while the external surface tension (γ_ext_) relates to the leaflet in contact with the external medium.

The morphology of an LD artificially inserted inside a bilayer is governed by the ratio of the surface tensions between the membrane bilayer and the droplet monolayer (Chorlay and Thiam, 2018). A high surface tension of the bilayer results in droplets with a lens-like morphology, whereas a lower bilayer surface tension leads to round-shaped droplets. However, if the surface tensions of the external and internal monolayer are equivalent, the droplet is positioned in the middle of the bilayer.

The direction of budding of a droplet is determined by an asymmetry between the luminal and cytosolic surface tensions (Chorlay and Thiam, 2018). When γ_int_ > γ_ext_, the droplet buds towards the exterior (Figure 2B; droplets on the right-hand side). Conversely, when γ_int_ < γ_ext_, the droplet buds towards the luminal side of the membrane (Figure 2B; droplets on the left-hand side). Finally, when γ_int_ = γ_ext_, the droplet fails to emerge, and stays embedded in the bilayer (Figure 2B; droplet in the middle). These findings indicate that in cells, for LDs to emerge towards the cytosol, the surface tension of the cytosolic leaflet of the ER membrane should be lower than that of the luminal leaflet.

The insertion of proteins or lipids into a lipid bilayer *in vitro* reduces surface tension by enhancing surface coverage, thereby facilitating proper droplet budding (Rangamani et al., 2014; Chorlay et al., 2019). A molecular dynamics study of a droplet embedded in a bilayer revealed that the addition of PC to one leaflet triggers the emergence of the droplet towards this side of the leaflet (Chorlay et al., 2019). In yeast, the addition of oleic acid to a growth medium depleted of inositol results in an increased number of LDs within cells (Chorlay et al., 2019). However, under these conditions, a higher number of LDs show large aera of contact with the ER membrane, indicating that these LDs fail to fully emerge from the ER. The supplementation of inositol to this medium boosts phospholipid synthesis by 30%, predominantly increasing PI levels, allowing for a higher number of LDs and reducing their area of contact with the ER. Similarly, in *Drosophila* cells, the depletion of CCT1, homolog of the yeast Pct1, the rate-limiting enzyme for PC synthesis through the Kennedy pathway, results in a higher number of LDs having a larger aera of contact with the ER membrane. Therefore, during LD formation, phospholipid replenishment on the cytosolic leaflet is important to reduce its membrane tension and thus promotes the proper emergence of LDs. In addition, the asymmetric insertion of proteins may contribute to decreasing surface tension by enhancing surface coverage of the cytoplasmic leaflet, thereby, wedge-shaped proteins, such as reticulons, may facilitate LD budding (Rangamani et al., 2014; Chorlay et al., 2019; Ferreira and Carvalho, 2021).

Interestingly, not all phospholipid species contribute equally to LD emergence (Table 1). The phospholipid composition of a membrane thus has an important impact on its surface tension (Ben M’barek et al., 2017). In addition, the neutral lipid composition of the LD core also affects the efficiency of droplet budding. For instance, PI has the tendency to decrease surface tension and effectively promotes the emergence of TAG-, TAG/STE-, and squalene-containing droplets (Ben M’barek et al., 2017). Conversely, PE and PA do not sufficiently decrease surface tension, hindering the proper budding of a LDs, independently of their neutral lipid composition. PC effectively supports the budding of TAG containing droplets but fails to facilitate the emergence droplets filled with TAG/STE or squalene (Ben M’barek et al., 2017). In a bilayer enriched in PE, neutral lipids tend to accumulate more between the bilayer leaflets than to condensate within the droplet. The addition of PC to such a bilayer reduces the retention of neutral lipids within the bilayer, promoting neutral lipid condensation and droplet emergence (Ben M’barek et al., 2017). Consequently, the combination between phospholipid and neutral lipid composition is decisive for the budding state of LDs.

**TABLE 1:** The lipid composition plays a crucial role in LD emergence by influencing both surface tension and membrane curvature.

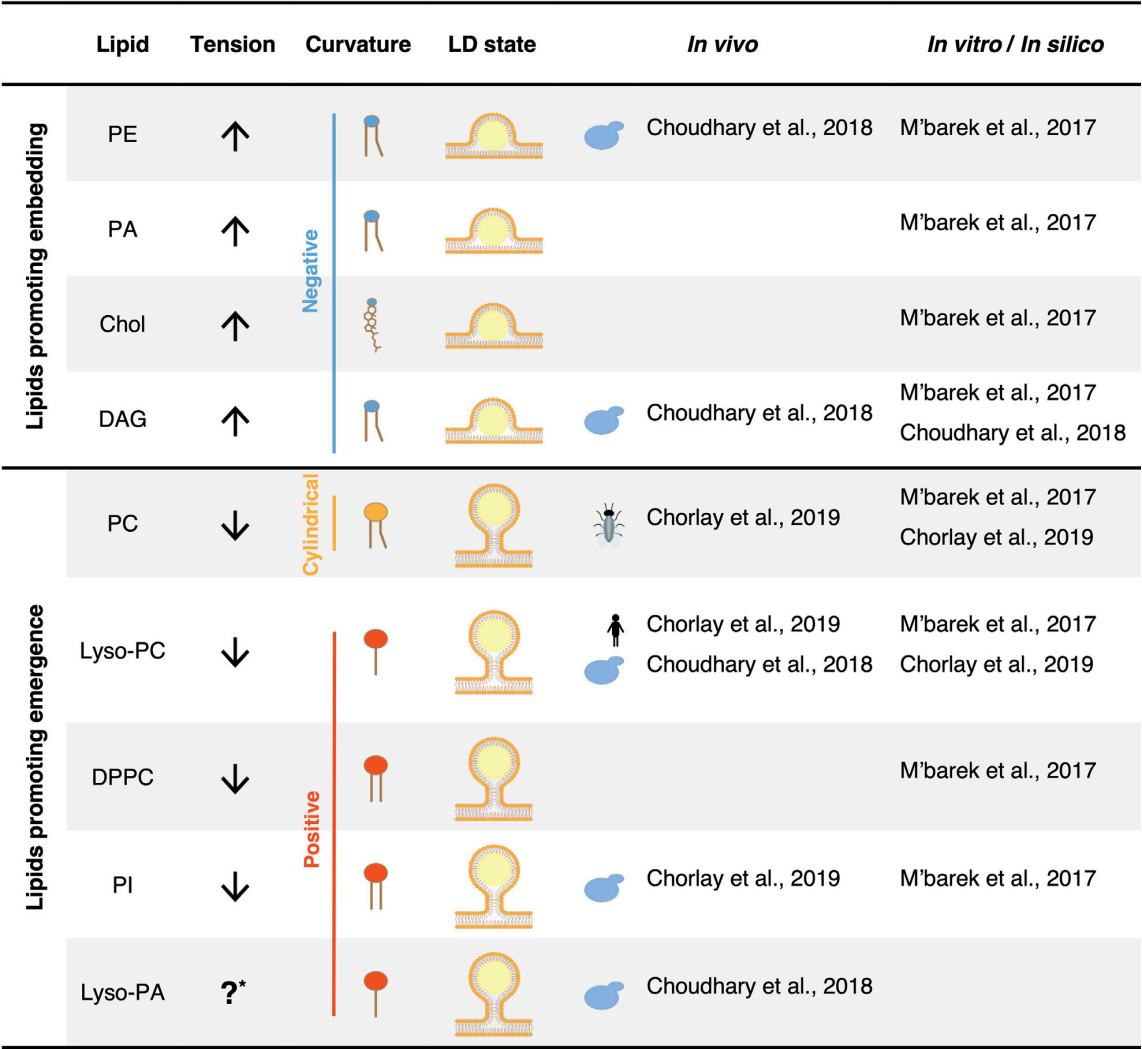									

*Abbreviations: Chol, cholesterol; DPPC, 1,2-dipalmitoyl-sn-glycero-3-phosphocholine; ? (uppercase1), no data available.

Taken together, these findings indicate that the phospholipid composition of a bilayer membrane affects its surface tension and, consequently, its elasticity. Interestingly, the two leaflets of a bilayer have different lipid compositions (Janmey and Kinnunen, 2006), which is believed to establish an asymmetry in the surface tension between the leaflets, enabling the emergence of the droplet towards the cytosol (Chorlay and Thiam, 2018). *In vivo*, the replenishment of specific phospholipids and the insertion of curvature promoting proteins into the cytosolic leaflet of the ER could serve as mechanisms to control and promote the budding of LDs towards the cytosol (Rangamani et al., 2014; Ben M’barek et al., 2017; Chorlay et al., 2019). Conversely, a deficiency in phospholipid synthesis or an aberrant phospholipid composition could promote the embedding of droplets within the ER membrane (Ben M’barek et al., 2017; Chorlay et al., 2019).

## 4 Phospholipids affect membrane curvature and LD budding

**FIGURE 3:**
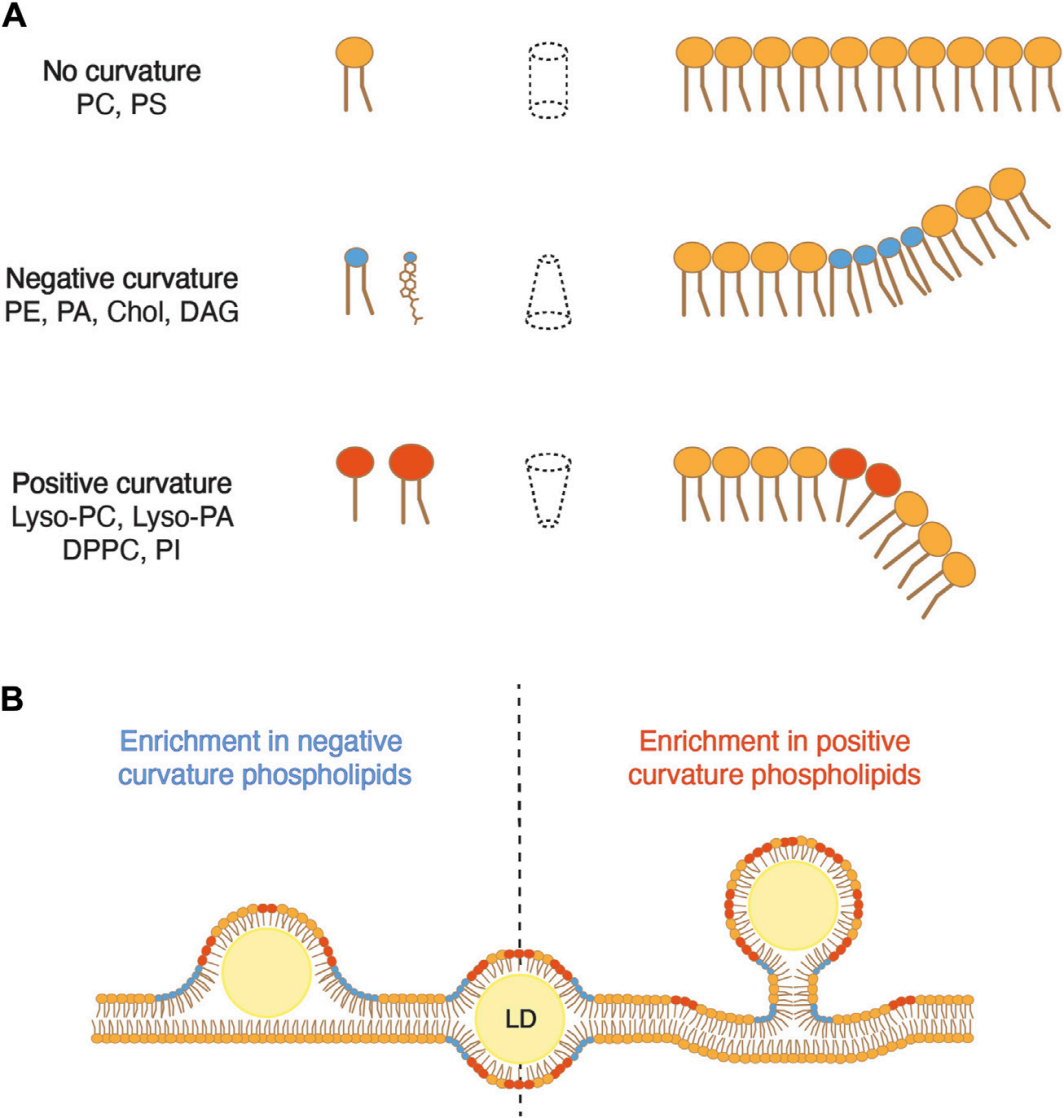
Membrane curvature can either promote or inhibit the emergence of LDs. **(A)** The dynamic shape of a phospholipid is determined by the relative size of the diameter of its polar head compared to that of its hydrophobic tails. When these diameters are comparable, the lipid occupies a cylindrical space, resulting in negligible membrane curvature. However, when the hydrophobic tail exceeds the diameter of the polar head, the lipid adopts an inverted cone shape, inducing negative membrane curvature. Conversely, when the polar head is larger than the hydrophobic tail, the lipid forms a cone shape, promoting positive curvature. PC, phosphatidylcholine; PS, phosphatidylserine; PE, phosphatidylethanolamine; PA, phosphatidic acid; Chol, cholesterol; DAG, diacylglycerol; Lyso-PC, lyso-phosphatidylcholine; Lyso-PA, lyso-phosphatidic acid; DPPC, dipalmitoyl phosphatidylcholine; PI, phosphatidylinositol. **(B)** An excess of phospholipids that induce negative curvature promotes the embedding of an LD in the membrane, whereas an excess of lipids inducing positive curvature promotes droplet emergence.

Beyond surface tension, the phospholipid composition of the ER membrane also influences membrane curvature, another parameter that regulates the efficiency of droplet budding. The dynamic shape of a phospholipid is characterized by the size of its polar headgroup in comparison that that of its acyl chains (Figure 3A; Table 1). Phospholipids with polar headgroups larger than the width of their acyl chains, such as PI or lyso-lipids in general, adopt the shape of an inverted cone and thereby induce positive membrane curvature. Conversely, phospholipids with a small polar headgroup compared to the width of their hydrophobic tail, such as PE or DAG, are cone-shaped and thus induce negative membrane curvature. Finally, phospholipids with similar polar width of their headgroup and acyl chains, such as PC and PS, adopt a cylindrical shape and do not induce a particular curvature of the bilayer membrane.

Modeling of LD formation from ER tubules suggests that elevated levels of DAG, which induces negative curvature, promotes the embedded state of the droplet (Choudhary et al., 2018). In yeast cells, DAG is synthesized by dephosphorylation of PA by the PA phosphatase Pah1. DAG can then be phosphorylated by the DAG kinase Dgk1 to regenerate PA. Deletion of *DGK1* significantly increases ER DAG levels, resulting in a higher number of embedded LDs compared to that of wild-type cells (Choudhary et al., 2018). Interestingly, cells deleted for *SCS3* and *YFT2* exhibit increased DAG levels in the ER membrane, notably accumulating at sites of LD biogenesis, and they also show an elevated number of embedded LDs (Choudhary et al., 2018). The deletion of *NEM1*, an activator of Pah1, in *scs3∆ yft2∆* double mutant cells, not only restores ER DAG levels but also facilitates the emergence of LDs. Comparable to DAG, PE is also a phospholipid that induces negative membrane curvature. PE undergoes methylation by two distinct enzymes, Cho2 and Opi3, to yield PC. The deletion of *CHO2* considerably increases the proportion of PE in phospholipids and correlates with a higher proportion of LDs that remain embedded in the ER membrane (Choudhary et al., 2018). Conversely, the addition of lyso-PC or lyso-PA, both promoting positive curvature, restores the emerged state of droplets in *scs3∆ yft2∆* cells. Taken together, these results indicate that a locally increased proportion of phospholipids that promote negative curvature in the ER membrane impedes proper budding of LDs, while phospholipids inducing positive curvature promote the emergence of droplets (Figure 3B).

The hypothesis that positive membrane curvature facilitates LD budding is supported by the observation that LD assembly primarily occurs at ER tubules (Santinho et al., 2020). In mammalian cells, ER tubules are enriched in seipin, a protein that promotes nucleation of neutral lipids and affects LD number and size. Even in the absence of seipin, LD formation still takes place and is correlated with the abundance of ER tubules. This is likely because the critical concentration for the nucleation of neutral lipids in curved areas of the membrane is lower than in flat areas. Specifically, the presence of diffused TAG is less favorable in the region with high membrane curvature, prompting it to nucleate into oily lenses, the precursors of LDs. However, STE does not exhibit the same characteristics as TAG, suggesting that the assembly of TAG- and STE-containing LDs might differ based on their neutral lipid composition (Renne et al., 2022).

In line with these findings, FITM-2, a *Caenorhabditis elegans* homolog of FIT2, was identified in a screen for mutants with impaired ER morphology (Chen et al., 2021). Moreover, FIT2 interacts with tubule-promoting proteins that localize at LD biogenesis sites in mammalian cells (Chen et al., 2021). Similar to FIT2, the loss of these tubule-promoting proteins results in fewer/smaller LDs, indicating that ER tubules provide a suitable curvature for promoting the initial step of LD formation (Chen et al., 2021).

## 5 Possible functions of FIT2 *in vivo*

This review underscores the critical function of the lipid composition on two key parameters affecting LD emergence, the membrane surface tension and membrane curvature. Lipids on the ER cytosolic leaflet that induce positive curvature and reduce surface tension promote the emergence of LDs. Conversely, lipids that induce negative curvature and increase surface tension favor the embedded state of droplets.

Interestingly, the lipid composition seems to be essential for several distinct steps of LD biogenesis, including the nucleation of neutral lipids in the ER bilayer. This process involves the de-mixing of TAG in the bilayer and its condensation to an oil lens, which subsequently grows and emerges into a mature LD. DAG, PE and cholesterol facilitate nucleation by reducing the diffusion of TAG within the bilayer (Zoni et al., 2021). However, during the emergence of LDs, these lipids favor the embedded state (Ben M’barek et al., 2017; Choudhary et al., 2018). These observations underscore the importance of a proper, local, and possibly dynamic composition of lipids at sites of LD biogenesis.

**FIGURE 4:**
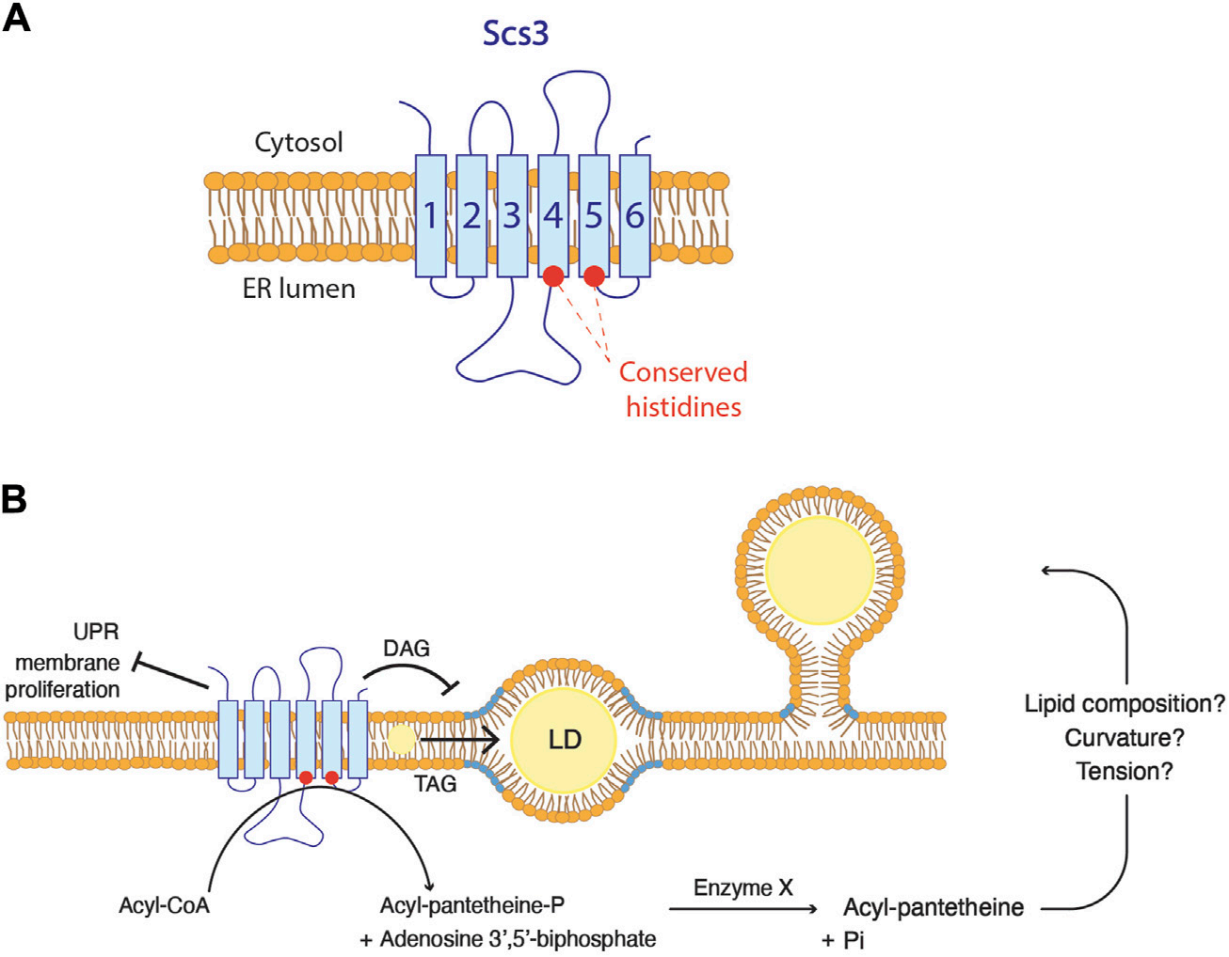
FIT2 proteins function in lipid droplet biogenesis **(A)** FIT2 homologs are composed of six transmembrane domains. The two conserved histidine residues are localized at the ER luminal leaflet. **(B)** Possible *in vivo* functions of FIT2 proteins. FIT2 decreases diacylglycerol (DAG) levels (in blue) at LD biogenesis sites, which might be a key for LD emergence. In addition, FIT2 participate in the partitioning of triacylglycerol (TAG) into LDs, allowing their proper expansion. Finally, FIT2 is involved in other processes, including ER stress. The activity of FIT2 to cleave acyl-CoA *in vitro* and how this might relate to LD emergence is indicated.

The embedded state of droplets observed in FIT2-deficient cells, suggests a plausible role of FIT2 in regulating the phospholipid composition at LD biogenesis site, thereby facilitating their proper emergence. Given that the highly conserved putative catalytic residues of FIT2 are located on the luminal side of the ER membrane, it is likely that FIT2 acts on lipids located in the luminal leaflet (Figure 4A) (Hayes et al., 2017). So far, the proposed diphosphatase activity of FIT2 has not yet been observed *in vivo*. The cleavage of acyl-CoA by FIT2 results in the formation of acyl 4′-phosphopantetheine, which is further dephosphorylated by an uncharacterized enzyme into acyl pantetheine. The formation of acyl pantetheine could potentially impact membrane properties, including its lipid composition, and hence its curvature and surface tension, facilitating the emergence of LDs towards the cytosol (Figure 4B). However, this possibility awaits experimental validation.

In *scs3∆ yft2∆* cells, DAG accumulates at LD biogenesis sites, suggesting that yeast FIT2 homologs are crucial to reduce this pool of DAG (Figure 4B) (Choudhary et al., 2018). *In vitro*, FIT2 interacts with both DAG and TAG, and binding efficiency correlates with the LD size (Gross et al., 2011). While it could be speculated that FIT2 is involved in TAG production, no changes in TAG synthesis are observed in FIT2 mutants (Kadereit et al., 2008). Even though the kinetics of TAG synthesis remain unchanged, the overall quantity of TAG is indeed increased in cells overexpressing FIT2 and decreased in cells defective for FIT2 (Kadereit et al., 2008; Agrawal et al., 2019). These findings suggest that FIT2 is not directly implicated in TAG biosynthesis but rather plays a role in the partitioning of TAG into LDs (Figure 4B). Conversely, FIT2 does not bind to cholesteryl oleate *in vitro*, nor does its overexpression induce an overall increase in cholesterol levels in mouse liver (Kadereit et al., 2008; Gross et al., 2011). These results indicate that the function of FIT2 is associated with the metabolism of phospholipids rather than that of sterols.

As mentioned above, *scs3∆ yft2∆* cells show accumulation of DAG at LD biogenesis sites (Choudhary et al., 2018). This is based on the localization of DAG using an ER-DAG sensor, composed of the C1 domains of Protein Kinase D fused to GFP and the transmembrane domain of Ubc6, a tail-anchored ER protein (Choudhary et al., 2018). This ER-DAG sensor is localized on the cytoplasmic side of the ER membrane. One hypothesis suggests that FIT2 may play a role in retaining DAG on the luminal side of the ER membrane. This retention could potentially contribute to establishing a lipid composition on the cytosolic leaflet that promotes the proper emergence of droplets. In the absence of FIT2, DAG localized on the luminal leaflet may flip to the cytosolic leaflet, favoring the embedded state of LDs.

On the other hand, DAG localized at LD biogenesis site could potentially be utilized for PC synthesis through the Kennedy pathway, which occurs at the cytosolic leaflet of the ER. This process would decrease the surface tension of the cytosolic leaflet, prompting the budding toward this side. Supporting this hypothesis, the induction of LD biogenesis in fly cells leads to the translocation of CCT1 from the nucleus to LDs until induction ceases (Krahmer et al., 2011). However, this PC synthesis through this pathway occurs at the cytosolic leaflet. In this case, FIT2 might be involved in flipping DAG or a derivative of it from the luminal to the cytosolic leaflet, thereby enabling the replenishment of phospholipids specifically on the cytosolic leaflet.

We have previously discussed that yeast cells expressing a mutant version of Scs3, in which the conserved histidine residues were replaced, exhibit inositol auxotrophy. Thus, these cells experience growth defects when cultivated without inositol, and these defects are exacerbated by the addition of choline to the medium. Exogenous choline serves as a substrate for PC synthesis through the Kennedy pathway. *Scs3∆* cells cultivated without inositol have higher PC levels. This phenotype is rescued by the deletion of Pct1, the rate-limiting enzyme of the Kennedy pathway, suggesting an upregulation of PC synthesis through this pathway in *scs3∆* cells (Yap et al., 2020). In addition, the overexpression of *NTE1*, a serine esterase responsible for degrading PC, rescues the growth defects observed in *scs3∆* cells on media lacking inositol irrespective of whether choline is present or not (Fernández-Murray et al., 2009). This underscores a mis-regulation of phospholipid synthesis in FIT2-depleted cells. Furthermore, *nte1∆* cells display growth defects on inositol-depleted media, but these defects are alleviated when cells are deficient for PC Kennedy enzymes (Cki1, Pct1 and Cpt1), highlighting the importance of PC turnover even in the absence of choline supplementation (Fernández-Murray et al., 2009).

Another notable phenotype associated with FIT2 dysfunction is an abnormal morphology of the ER membrane, marked by a dilatation of the luminal space and membrane proliferations, resulting in onion-like structures frequently referred to as ER whorls (Chorlay et al., 2019; Becuwe et al., 2020). The impact of FIT2-deficient cells on membrane morphology is specific for the ER, as there are no changes in the morphology of lysosomes, peroxisomes, or the Golgi apparatus (Becuwe et al., 2020). This aberrant ER morphology points towards an unresolved ER stress, as it resembles the phenotype of mutants of the unfolded protein response (UPR) (Schuck et al., 2009). Consistently, markers of ER stress are moderately upregulated in FIT2-deficient cells (Becuwe et al., 2020; Bond et al., 2023). In mice pancreatic β-cells, direct palmitoylation of FIT2 following saturated fat addition results in its degradation and is correlated with expression of ER stress markers (Zheng et al., 2022). In yeast, the accumulation of unfolded proteins in the ER is detected by the transmembrane sensor Ire1 (Cox et al., 1993). In support of a deficiency in the UPR in FIT2-deleted cells, a *scs3∆ ire1∆* double mutant is lethal, and *scs3∆* cells display an increased sensitivity to DTT, a reducing agent that induces protein misfolding (Moir et al., 2012; Yap et al., 2020). Importantly, the resistance of wild-type cells to DTT-induced stress requires a remodeling of the lipidome (Volmer and Ron, 2015; Reinhard et al., 2020). Collectively, these findings support the essential role of FIT2 in enabling cells to adapt their lipid composition to changing cellular conditions (Figure 4B).

Supporting an essential role for FIT2 in adapting the lipid composition of the ER, a synthetic genetic array showed genetic interactions between Scs3/Yft2 and numerous genes involved in lipid metabolism (Moir et al., 2012). The interactions encompass genes related to phospholipid metabolism, such as PI or PC synthesis genes, as well as genes involved in the metabolism of more complex lipids like sphingolipids. Additionally, a hepatic lipidomic analysis of hepatocyte-specific FIT2 deficient mice reported alterations in various lipid levels, including specific acyl-CoA, neutral lipid, phospholipid, and sphingolipid species (Bond et al., 2023). Taken together, these finding suggest that FIT2’s function in lipid metabolism extends beyond phospholipids and neutral lipids. Consequently, FIT2 might be required to globally regulate the ER lipid composition, enabling the cell to adapt to changing environments.

In conclusion, FIT2 function provides an intricate connection between ER homeostasis and the biogenesis of LDs. Through regulating the lipid composition of the ER membrane, FIT2 is suggested to play a crucial role in facilitating the adaptation of the ER membrane to the synthesis and nucleation of neutral lipids and the emergence of LDs. Additionally, FIT2 may be involved in alleviating other ER stresses, such as the accumulation of unfolded proteins. Consequently, FIT2 emerges as an important factor required for ER adaptation, extending beyond its primary association with LD biogenesis. Further exploration of the function of FIT2 in these processes is anticipated to provide valuable insights into the etiology of complex syndromes and metabolic disorders that are intricately connected to pathological alterations of lipid metabolism.

## 6 Significance statement

The intracellular storage of metabolic energy in lipid droplets (LDs) is a fundamental process vital for cellular homeostasis. Understanding the intricate mechanisms governing LD biogenesis has gained significant attention due to their implications in prevalent metabolic disorders such as type 2 diabetes and obesity. Among the proteins involved, Fat storage-Inducing Transmembrane 2 (FIT2) stands out for its pivotal role in LD formation. FIT2 influences the emergence step of LD biogenesis, as in FIT2 mutant cells LDs remain embedded within the endoplasmic reticulum (ER) membrane instead of properly emerging into the cytosol. While *in vitro* studies have demonstrated FIT2’s ability to bind di- and triacylglycerol (DAG/TAG) and cleave acyl-CoA, the translation of these functions to the observed LD embedding in FIT2 deficient cells remains to be fully elucidated. This article seeks to narrow this gap by reviewing FIT2’s role in LD emergence. We highlight the factors affecting LD emergence, particularly focusing on membrane curvature, surface tension, and lipid composition, and their interplay with FIT2. Additionally, we explore hypotheses proposing how FIT2 may locally modulate lipids at sites of LD emergence, shedding light on its intricate function within cellular lipid metabolism.

## Figures and Tables

**Table:** 

**CCT1**	Choline-phosphate cytidylyltransferase 1
**Cho2**	Choline requiring 2
**Cki1**	Choline kinase 1
**Cpt1**	Choline phosphotransferase 1
**DAG**	Diacylglycerol
**Dgk1**	Diacylglycerol kinase 1
**DTT**	Dithiothreitol
**ER**	Endoplasmic reticulum
**FIT1**	Fat storage-Inducing Transmembrane 1
**FIT2**	Fat storage-Inducing Transmembrane 2
**Ire1**	Inositol requiring 1
**LD**	Lipid droplet
**Nem1**	Nuclear envelope morphology 1
**Nte1**	Neuropathy target esterase 1
**Opi1**	Overproducer of inositol 1
**Opi3**	Overproducer of inositol 3
**PA**	Phosphatidic acid
**Pah1**	Phosphatidic acid phosphohydrolase 1
**PC**	Phosphatidylcholine
**Pct1**	Phosphocholine cytidylyltransferase
**PE**	Phosphatidylethanolamine
**PI**	Phosphatidylinositol
**PS**	Phosphatidylserine
**Scs3**	Suppressor of choline sensitivity 3
**STE**	Steryl ester
**TAG**	Triacylglycerol
**Ubc6**	Ubiquitin-conjugating 6
**UPR**	Unfolded protein response
**Yft2**	Yeast fat storage-inducing transmembrane 2
**Γext**	External surface tension
**Γint**	Internal surface tension
